# Daptomycin associated eosinophilic pneumonia: case report and differential diagnoses

**DOI:** 10.1080/20009666.2018.1475188

**Published:** 2018-06-12

**Authors:** Sijan Basnet, Niranjan Tachamo, Rashmi Dhital, Biswaraj Tharu

**Affiliations:** a Department of Medicine, Reading Hospital and Medical Center, West Reading, PA, USA; b Maharajgunj Medical Campus, Tribhuvan University, Kathmandu, Nepal

**Keywords:** Daptomycin, eosinophilic pneumonia, adverse effect

## Abstract

Daptomycin is a bactericidal antibiotic approved for treatment of gram-positive skin and soft tissue infections. We present a case of an 89-year-old man who presented with fever, shortness of breath and nonproductive cough on week 4 of starting daptomycin for infective endocarditis. Computerized tomography scan showed bilateral interstitial infiltrates predominantly affecting the lower lobes. He also had peripheral eosinophilia of 6%. He was diagnosed with eosinophilic pneumonia secondary to daptomycin use. His symptoms improved with discontinuation of daptomycin and initiation of corticosteroids. Clinical correlation of pneumonia-like presentation with recent use of daptomycin should make physicians rule out this rare adverse effect for early institution of correct treatment.

## 1. Introduction

Daptomycin is a lipopeptide antibiotic approved by the Food and Drug Administration (FDA) for treatment of skin and soft tissue infections by *Staphylococcus aureu*s (methicillin-susceptible or resistant strains), *Streptococcus* species and vancomycin-susceptible *Enterococcus faecalis*[1,2] Use of daptomycin has been increasing recently as an effective treatment alternative to vancomycin [3]. Subsequently, rare adverse effects like eosinophilic pneumonia are being reported more frequently. Daptomycin associated eosinophilic pneumonia (DAEP), although rare, can mimic other pneumonia resulting in delayed diagnosis and management, and hence has the potential to cause serious morbidity and mortality [1].

## 2. Case description

An 89-year-old man presented to the emergency department (ED) with a history of fever, nonproductive cough and exertional shortness of breath for a few days. He was started 4 weeks previously on intravenous daptomycin for 6 weeks for coagulase-negative staphylococcal bacteremia and infective endocarditis of mitral valve following a urinary procedure. Daptomycin was chosen in this patient for once daily dosing. He was a nonsmoker with no history of chronic obstructive pulmonary disease or asthma. In the ED, he was afebrile (temperature 99.8°F) with a heart rate of 77 bpm, blood pressure of 129/74 mm Hg and respiratory rate of 29 breaths per minute. His initial oxygen saturation was 72% on room air and required high flow oxygen to maintain his oxygen saturation. Chest examination revealed vesicular breath sounds with diffuse rales. Cardiac auscultation was normal. The rest of the physical examination was unremarkable.

**Figure 1.**
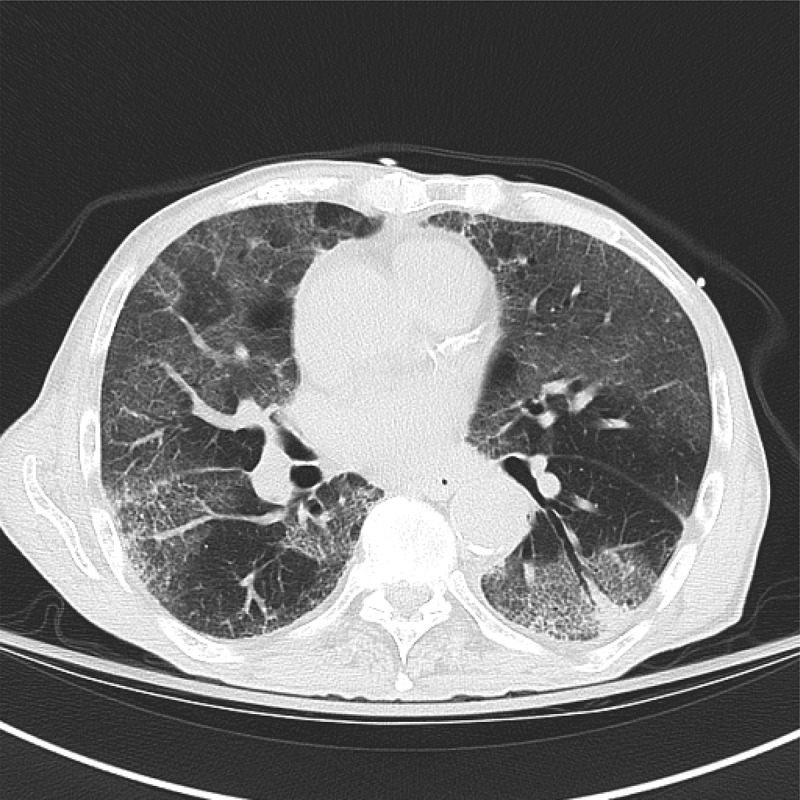
CT scan of chest showing bilateral diffuse ground glass opacity of lung parenchyma on presentation.

Pertinent labs included white count of 7900/µl with peripheral eosinophilia of 6% (reference range: 0.0–6.0%), Eosinophil count 500/µl (reference range: 150–300/uL), C-reactive protein 27.95 mg/dL (reference range: <1.00 mg/dL), erythrocyte sedimentation rate 54 mm/h (reference range: 0–15 mm/h) and brain natriuretic peptide 102 pg/mL (reference range: 0–100 pg/mL). Electrocardiogram was unremarkable. Chest X-ray demonstrated hazy bilateral airspace opacification predominantly in the lower lobes. Computerized tomography (CT) scan of the chest revealed bilateral lower lobe ground-glass opacification with interlobular septal thickening (Figure 1). *Streptococcus pneumoniae* and *Legionella* urinary antigens were negative. ANA, Rheumatoid factor, ANCA and respiratory pathogen panel were negative. Patient lives in Pennsylvania and denied travel outside the USA. Stool ova and parasites done were negative. Urine drug screen was not done but patient denied substance abuse. He was not on any over the counter pain medications. Blood and urine cultures done during hospital stay were negative.

With concern for DAEP, daptomycin was discontinued. Intravenous methylprednisolone at 60 mg every 6 h was started. Daptomycin was switched to intravenous vancomycin to complete the 6-week antibiotic course for infective endocarditis. He was also started on intravenous cefepime and metronidazole (2-week course) for broad-spectrum coverage of pneumonia. Resolution of eosinophilia (0.8%) with the appearance of neutrophilic leucocytosis (92.3% neutrophils with WBC of 12,500/µl) were noted. With improvement of his respiratory status, his methylprednisolone was stopped, and he was started on prednisone taper for 6 weeks. He was also started on prophylactic sulfamethoxazole-trimethoprim 800/160 mg two times daily until his prednisone would drop down to 20 mg daily. High flow oxygen was weaned off to nasal cannula oxygen.

**Figure 2.**
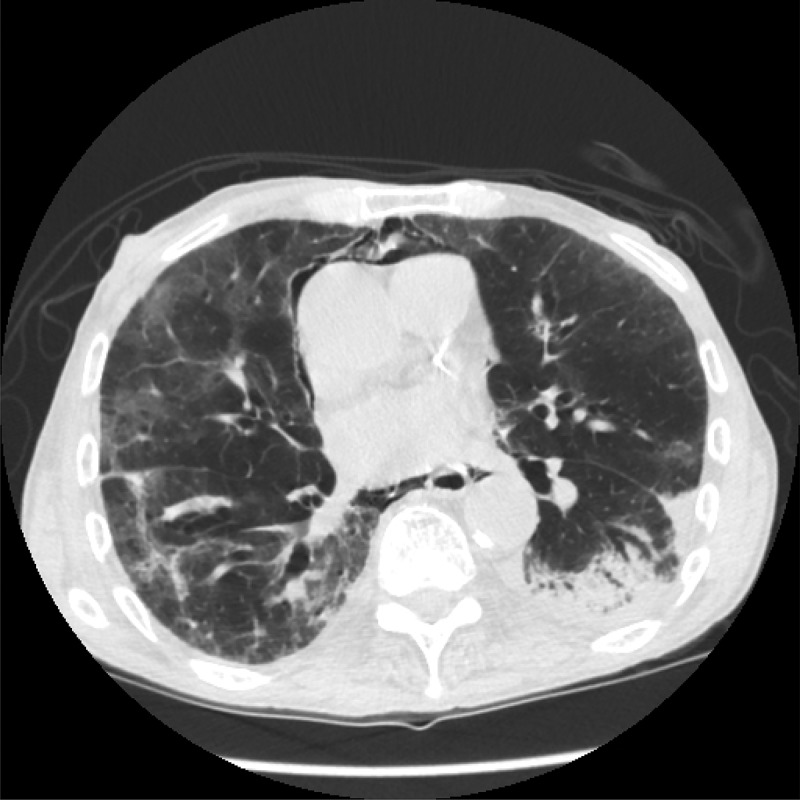
CT scan of chest showing improved opacity of lung parenchyma 2 weeks after discontinuation of daptomycin.

Repeat CT chest (Figure 2) 2 weeks later revealed partial clearing of the diffuse ground-glass opacities. His respiratory status had improved, and he was maintaining saturation in low 90s on 2–3-L oxygen via nasal cannula. He was discharged home on prednisone taper, prophylactic sulfamethoxazole-trimethoprim, and supplemental oxygen. Unfortunately, he was lost to follow-up.

## 3. Discussion

DAEP was first reported in 2007. DAEP is characterized by eosinophilic infiltration of the lung parenchyma ranging from asymptomatic infiltrates to acute respiratory distress syndrome necessitating mechanical ventilation [4]. It is believed that parenchymal damage secondary to destruction of pulmonary surfactant and induction of allergic reaction by daptomycin causes DAEP [4–7].

DAEP is characteristically seen in elderly males and usually manifests as a febrile illness lasting a few weeks and is accompanied by constitutional symptoms, dyspnea and nonproductive cough [4,8]. Kim et al developed a case definition for DAEP and classified it as definite, probable, possible and unlikely based on fulfillment of criteria. Diagnosis is ‘definite’ if there is a history of exposure to daptomycin, fever, dyspnea requiring oxygen or mechanical ventilation, pulmonary infiltrates identified on chest X-ray or CT scan, bronchoalveolar lavage (BAL) with >25% eosinophils and clinical improvement with discontinuation of daptomycin. Diagnosis is ‘probable’ if there is a history of daptomycin exposure, dyspnea requiring oxygen or mechanical ventilation, pulmonary infiltrates identified on chest X-ray or CT scan, peripheral eosinophilia and clinical improvement following daptomycin withdrawal [4], as in our case. We did not do a bronchoscopy as our patient was frail. Initial diagnostic studies may show hypoxemia, infiltrates on chest imaging and variably, peripheral eosinophilia [4]. Pulmonary eosinophilic infiltrates or BAL eosinophilia is a cornerstone for diagnosis [2,9]. Discontinuation of daptomycin with or without corticosteroid is the mainstay of management [4,8]. Corticosteroid is believed to rapidly reverse the inflammation and thereby improve the hypoxia [8]. Patients are usually started on intravenous methylprednisone 60–125 mg every 6–8 h, transitioned to oral prednisone 40–60 mg daily with clinical improvement and is tapered over 2–6 weeks to avoid relapse [8,10].

## 4. Differential diagnosis

Conditions that can have a similar presentation of dyspnea and bilateral ground glass pulmonary infiltrates include pneumonia, hypersensitivity pneumonitis, alveolar proteinosis and cryptogenic organizing pneumonia. The patient did not have constitutional symptoms, sputum production with negative pneumococcal and Legionella urinary antigens making pneumonia unlikely. *Pneumocystis jirovecii* pneumonia without constitutional symptoms, history of exposure to HIV or high-risk behavior can be ruled out. Alveolar proteinosis is a rare diagnosis which is uncommon with no history of smoking. Lastly, cryptogenic organizing pneumonia is characteristically associated with a change in location of consolidation which was absent in our patient on serial imaging.

## 5. Conclusion

DAEP is an uncommonly observed adverse effect of daptomycin. Its incidence will increase with increasing use of daptomycin. Clinicians must be aware of this adverse effect and presentation for early discontinuation of the medication and institution of corticosteroids.

## Key points

1. Eosinophilic pneumonia is a rare side effect of daptomycin.
2. It presents as febrile illness, nonproductive cough and shortness of breath within a few weeks of initiation of daptomycin.
3. Diagnosis is ‘definite’ if there is a history of exposure to daptomycin, fever, dyspnea requiring oxygen or mechanical ventilation, pulmonary infiltrates identified on chest X-ray or CT scan, bronchoalveolar lavage (BAL) with >25% eosinophils and clinical improvement with discontinuation of daptomycin.
4. Discontinuation of daptomycin and corticosteroids are the primary treatment measures.
